# Phase II trial of gemcitabine, epirubicin and granulocyte colony-stimulating factor in patients with advanced pancreatic adenocarcinoma

**DOI:** 10.1038/sj.bjc.6690600

**Published:** 1999-08

**Authors:** W Scheithauer, G V Kornek, M Raderer, M Hejna, J Valencak, J Miholic, E Kovats, F Lang, J Funovics, E Bareck, D Depisch

**Affiliations:** Division of Oncology and Department of Internal Medicine I and Department of Surgery, Vienna University Medical School, Waehringer Guertel 18-20, Vienna, A-1090, Austria; Department of Surgery, Baden General Hospital, Wimmergasse 19, Baden, A-2500, Austria; Department of Surgery, Neunkirchen General Hospital, Peischinger Strasse 19, Neunkirchen, A-2620, Austria; Department of Surgery, Stockerau General Hospital, Landstrasse 16-18, Stockerau, A-2000, Austria; Department of Surgery, Wr. Neustadt General Hospital, Corvinusring 3-5, Wr. Neustadt, A-2700, Austria

**Keywords:** pancreatic cancer, chemotherapy, gemcitabine, epirubicin

## Abstract

Although the novel cytidin analogue gemcitabine has shown superior anti-tumour activity than 5-fluorouracil in advanced pancreatic cancer, further improvements of therapeutic results are warranted. This goal might be achieved by combining gemcitabine with other active drugs. This trial evaluated the efficacy and tolerance of such a combination regimen with epirubicin and granulocyte colony-stimulating factor (G-CSF) in patients with metastatic disease. Seventy patients with metastatic pancreatic adenocarcinoma were enrolled in this multicentre trial. Patients received 4-weekly courses of a combination regimen consisting of epirubicin 60 mg m^−2^ given as intravenous bolus injection on day 1, gemcitabine 1000 mg m^−2^ infused over 30 min on days 1, 8 and 15, and G-CSF administered at 5 μg kg^−1^ day^−1^ subcutaneously from days 2–6 during each cycle. The efficacy of treatment was assessed by conventional measures, i.e. objective response, progression-free and overall survival, as well as by analysis of clinical benefit response (defined as ≥ 50% reduction in pain intensity, ≥ 50% reduction in daily analgesic consumption, and/or ≥ 20-point improvement in Karnofsky performance status that was sustained for ≥ 4 consecutive weeks). Of 66 patients evaluable for objective response, one achieved complete and 13 partial remissions, for an overall response rate of 21% (95% confidence interval (CI), 12–33%); 27 additional patients (41%) had stable and 25 (38%) increasing disease. The median time to progression was 3.8 months. Median survival was 7.8 months, and the probability of surviving beyond 12 months was 21.2%. Out of 60 patients with tumour-related symptoms, who were considered evaluable for clinical benefit response, 26 (43%) experienced significant palliation. The median time to achieve a clinical benefit response was 7 weeks, and its median duration was 22 weeks. Chemotherapy was well-tolerated with leukopenia/granulocytopenia representing the most common and dose-limiting side-effect. Gastrointestinal and other subjective toxicities were infrequent and generally rated minor. We conclude that the combination of gemcitabine, epirubicin and G-CSF seems to be an effective palliative treatment with only moderate toxic effects in patients with metastatic pancreatic adenocarcinoma. Our results in terms of objective and clinical benefit response, as well as survival seem to suggest an advantage over gemcitabine-monotherapy, though this remains to be confirmed in a randomized trial. © 1999 Cancer Research Campaign


*British Journal of Cancer* (1999) **80**(11), 1797–1802
© 1999 Cancer Research Campaign
Article no. bjoc.1999.0600

### Phase II trial of gemcitabine, epirubicin and granulocyte colony-stimulating factor in patients with advanced pancreatic adenocarcinoma

**W Scheithauer[1], GV Kornek[1], M Raderer[1], M Hejna[1], J Valencak[1], J Miholic[2], E Kovats[3], F Lang[4], J Funovics[5], E Bareck[6] and D Depisch[6]**

[1]Division of Oncology, Department of Internal Medicine I and [2]Department of Surgery, Vienna University Medical School, Waehringer Guertel 18-20, A-1090 Vienna, Austria; [3]Department of Surgery, Baden General Hospital, Wimmergasse 19, A-2500 Baden, Austria; [4]Department of Surgery, Neunkirchen General Hospital, Peischinger Strasse 19, A-2620 Neunkirchen, Austria; [5]Department of Surgery, Stockerau General Hospital, Landstrasse 16-18, A-2000 Stockerau, Austria; [6]Department of Surgery, Wr. Neustadt General Hospital, Corvinusring 3-5, A-2700 Wr. Neustadt, Austria

**Summary** Although the novel cytidin analogue gemcitabine has shown superior anti-tumour activity than 5-fluorouracil in advanced pancreatic cancer, further improvements of therapeutic results are warranted. This goal might be achieved by combining gemcitabine with other active drugs. This trial evaluated the efficacy and tolerance of such a combination regimen with epirubicin and granulocyte colony-stimulating factor (G-CSF) in patients with metastatic disease. Seventy patients with metastatic pancreatic adenocarcinoma were enrolled in this multicentre trial. Patients received 4-weekly courses of a combination regimen consisting of epirubicin 60 mg $m^{-2}$ given as intravenous bolus injection on day 1, gemcitabine 1000 mg $m^{-2}$ infused over 30 min on days 1, 8 and 15, and G-CSF administered at 5 μg $kg^{-1}$ $day^{-1}$ subcutaneously from days 2–6 during each cycle. The efficacy of treatment was assessed by conventional measures, i.e. objective response, progression-free and overall survival, as well as by analysis of clinical benefit response (defined as ≥ 50% reduction in pain intensity, ≥ 50% reduction in daily analgesic consumption, and/or ≥ 20-point improvement in Karnofsky performance status that was sustained for ≥ 4 consecutive weeks). Of 66 patients evaluable for objective response, one achieved complete and 13 partial remissions, for an overall response rate of 21% (95% confidence interval (CI), 12–33%); 27 additional patients (41%) had stable and 25 (38%) increasing disease. The median time to progression was 3.8 months. Median survival was 7.8 months, and the probability of surviving beyond 12 months was 21.2%. Out of 60 patients with tumour-related symptoms, who were considered evaluable for clinical benefit response, 26 (43%) experienced significant palliation. The median time to achieve a clinical benefit response was 7 weeks, and its median duration was 22 weeks. Chemotherapy was well-tolerated with leukopenia/granulocytopenia representing the most common and dose-limiting side-effect. Gastrointestinal and other subjective toxicities were infrequent and generally rated minor. We conclude that the combination of gemcitabine, epirubicin and G-CSF seems to be an effective palliative treatment with only moderate toxic effects in patients with metastatic pancreatic adenocarcinoma. Our results in terms of objective and clinical benefit response, as well as survival seem to suggest an advantage over gemcitabine-monotherapy, though this remains to be confirmed in a randomized trial.

**Keywords**: pancreatic cancer; chemotherapy; gemcitabine; epirubicin

Pancreatic adenocarcinoma, which is responsible for almost 5% of all cancer related deaths in the Western world (Parker et al, 1996), continues to be a major unresolved health problem. The large majority of patients present with disease that is beyond the scope of surgical cure, and their prognosis is extremely poor: in case of distant metastases, the median survival duration is generally less than 3 months (Schnall and Macdonald, 1996).

Single chemotherapeutic agents, as well as combination regimens, have shown only modest activity in this fatal disease, with response rates of the most active agents in the 10–20% range (Warshaw and Fernandez-del Castillo, 1992). Recently, gemcitabine, a novel nucleoside analogue with preclinical activity against a broad spectrum of solid tumours (Hertel et al, 1990), was evaluated in a multicentre trial of 44 patients with advanced pancreatic cancer (Casper et al, 1994). While only five objective responses (11%) were documented, the investigators noted frequent subjective symptomatic benefit, often in the absence of an objective response. In a subsequent randomized trial involving 126 previously untreated patients (Burris et al, 1997), gemcitabine was compared with 5-fluorouracil (5-FU). Patients treated with gemcitabine achieved modest but statistically significant improvements in response rate and median survival compared with those treated with 5-FU (5.6 vs 4.4 months). In addition, more clinically meaningful effects on disease-related symptoms (pain control, improvement in performance status and weight gain) were seen with gemcitabine than with 5-FU (24% vs 5%). Similar clinically beneficial effects were noted in patients who were treated with gemcitabine after experiencing disease progression while receiving 5-FU (Rothenberg et al, 1996). Although these recent encouraging results with gemcitabine are a step in the right direction, better treatments for pancreatic cancer are certainly needed. One possible approach to further improve therapeutic results may represent the combination of gemcitabine with other active cytotoxic drugs.

*Received 13 October 1998*
*Revised 3 February 1999*
*Accepted 20 February 1999*

*Correspondence to:* W Scheithauer

This multicentre phase II trial was performed to determine the anti-tumour activity of gemcitabine plus epirubicin in patients with advanced pancreatic cancer. The latter drug was chosen because of its documented activity in this disease (Wils et al, 1985; Kornek et al, 1995), and its potential drug synergism without (non-haematologic) cross toxicity (Lueftner et al, 1996; Garcia-Conde et al, 1997). To allow administration of adequate drug doses of both gemcitabine and epirubicin, and to prevent/counteract myelosuppression that was assumed to represent the dose-limiting toxicity (Lueftner et al, 1996), granulocyte colony-stimulating factor (G-CSF) using a convenient and cost-effective 5-day administration schedule was routinely used (Ribas et al, 1996). The objective of our trial was to determine the anti-tumour efficacy and tolerance of this combination regimen in patients with metastatic pancreatic adenocarcinoma. The former was assessed by conventional measures, i.e. objective response, time to progression and median survival, as well as by clinical benefit response analysis as previously described (Rothenberg et al, 1996; Burris et al, 1997).

### PATIENTS AND METHODS

### Patient selection

To be entered in this trial, all patients were required to have histologically or cytologically ascertained metastatic adenocarcinoma of the pancreas. Patients with resectable tumours as well as those with locally advanced, inoperable disease were not included in the study. All patients were required to have bidimensionally measurable disease, to be 75 years of age or younger, and to have an anticipated life expectancy of al least 3 months. Furthermore, patients were required to have a baseline Karnofsky performance status of at least 50, and to have adequate renal (serum creatinine level < 1.5 mg $dl^{-1}$), liver (total bilirubin level < 1.5 mg $dl^{-1}$ and transaminase levels less than two times the upper limits of normal) and bone marrow function (leucocyte count ≥ 4000 $\mu l^{-1}$, absolute granulocyte count ≥ 2000 $\mu l^{-1}$ and platelet count ≥ 100 000 $\mu l^{-1}$). In addition, all patients had to have normal pretreatment electrocardiograms (ECG) and echocardiograms (left ventricular ejection fraction of more than 50% and no wall motion abnormalities). Patients with a history of cardiovascular disease, any other serious or uncontrolled concurrent medical illness or with central nervous system metastases were not eligible for treatment, as were those who had undergone any prior palliative chemotherapy or radiotherapy. A minimum of 2 weeks was required to have elapsed in case of prior abdominal exploration or palliative surgery. Informed consent was obtained from all patients according to institutional regulations.

### Pretreatment and follow-up evaluation

Pretreatment evaluation included a complete medical history, physical examination, ECG, echocardiography and routine laboratory studies. The latter consisted of a complete blood count (CBC) with platelet and leucocyte differential count, and an 18-function biochemical profile. Imaging procedured included chest X-ray and computerized tomography of the abdomen. CBCs, differential counts and liver functional parameters were determined weekly, and complete biochemical profiles were assessed before each treatment cycle. Objective tumour assessments were performed at the end of every two cycles during chemotherapy and every 3 months after discontinuation of treatment. Echocardiography was repeated every 8–12 weeks during therapy.

### Treatment protocol

Chemotherapy consisted of epirubicin 60 mg $m^{-2}$ given as an intravenous (i.v.) bolus injection on day 1, gemcitabine 1000 mg $m^{-2}$ diluted in normal saline and administered i.v. over 30 min on days 1, 8 and 15, plus G-CSF administered at 5 $\mu g\ kg^{-1}\ day^{-1}$ subcutaneously from days 2–6 during each cycle. Treatment courses were repeated every 4 weeks, and continued in patients achieving objective response or stable disease until a total of six courses. Concomitant medications routinely administered before cytotoxic drug administration included 8 mg ondansetron plus 8 mg dexamethasone (the latter given only on day 1).

### Toxicity and dosage modification guidelines

Adverse reactions were evaluated according to World Health Organization (WHO) criteria (Milleret al, 1981). Chemotherapeutic drug doses were reduced by 25% in subsequent cycles if the lowest WBC (absolute granulocyte) count was less than 1000 $\mu l^{-1}$ (500 $\mu l^{-1}$), the lowest platelet count was less than 50 000 $\mu l^{-1}$, or if any severe (≥ WHO grade 3) nonhaematologic toxicity was observed in the previous cycle. Treatment could be delayed for up to 2 weeks if the WBC count was lower than 3000 $\mu l^{-1}$ and/or the platelet count lower than 75 000 $\mu l^{-1}$; prolonged administration of G-CSF was recommended in the former group of patients. Any patient who required more than 2 weeks for haematologic recovery was taken off the study.

### Assessment of objective and clinical benefit response

The primary efficacy end point was response rate. A complete response (CR) was defined as the disappearance of all clinical evidence of tumour for a minimum of 4 weeks during which time the patient was free of all symptoms related to cancer. Partial response (PR) was defined as a >50% decrease in the sum of the products of the longest perpendicular diameters of all measurable disease with no new lesions appearing and none progressing for at least 4 consecutive weeks. Patients were rated progressive (PD) if any new lesion appeared, or tumour size increased by 25% over pretreatment measurements, or in case of a deterioration in clinical status that was consistent with disease progression. Patients who failed to meet the criteria of CR, PR, or PD and who remained on study for at least 2 months were classified as having stable disease (SD). Two objective measurements that showed a response at at least 4-week intervals were required to confirm a patients' response; all tumour measurements in patients who responded were reviewed and confirmed by a reference radiologist. Secondary efficacy endpoints included the duration of response (measured from the onset of the best response to the date of disease progression), time to progression (TTP; calculated from the date of initiation of therapy to the date when progressive disease was first observed) and overall survival.

In addition to these 'objective study end-points', clinical benefit was evaluated in symptomatic patients as previously described (Rothenberg et al, 1996; Burris et al, 1997). Pain (computed as the mean of the pain intensity scores recorded daily by the patient on a 100 mm VAS, plus analgesic consumption (expressed as morphine equivalent mg per day) computed as the mean of the daily use indicated in a diary) and karnofsky performance status (assessed weekly by two independent observers with selection of the lower value if the scores differed) comprised the primary measures of

© 1999 Cancer Research Campaign clinical benefit and were assessed weekly. Weight change, also recorded weekly (and excluding patients who developed third-space fluid or required parenteral nutrition at any time during the study) was considered a secondary measure. To achieve an overall rating of positive clinical benefit response, patients had to be positive for at least one parameter (pain: a ≥ 50% improvement in pain intensity and/or a ≥ 50% decrease in analgesic consumption compared to baseline; Karnofsky performance status: a ≥ 20-point improvement over baseline; weight: increase by ≥ 7% over baseline) without being negative for any of the others (i.e. deterioration in pain intensity measurements and/or increase in analgesic consumption by any degree; worsening in performance status by ≥ 20 points over baseline). This improvement had to last for ≥ 4 weeks. The primary measures of pain and performance status were evaluated first; a patient who was rated stable on these primary measures (i.e. categorized neither as positive or as negative) could be classified as having achieved an overall clinical benefit response only if weight was positive. All other patients were classified as not having achieved clinical benefit response.

The duration of clinical benefit response was defined as the duration of the positive classification in case of a single component. If multiple components were positive, the duration of clinical benefit response was defined as the largest number of consecutive weeks during which there was a positive change for at least one of the components.

**Table 1** Pretreatment characteristics

| Characteristic | No. of patients (%) |
|---|---|
| Number of patients entered/eligible | 70/66 |
| Sex | |
| Male | 39 (59) |
| Female | 27 (41) |
| Median age in years (range) | 62 (32–75) |
| Karnofsky performance status | |
| 90–100 | 6 (9) |
| 70–80 | 33 (50) |
| 50–60 | 27 (41) |
| Prior surgery | |
| None | 26 (39) |
| Explorative laparotomy | 11 (17) |
| Palliative bypass | 15 (23) |
| Whipple or left resection | 14 (21) |
| Histological grade | |
| G1 | 4 (6) |
| G2 | 40 (61) |
| G3 | 22 (33) |
| Sites of metastases | |
| Liver | 46 (70) |
| Abdominopelvic mass | 60 (91) |
| Lung | 5 (8) |
| Extraabd.lymph nodes/soft-tissue | 4 (6) |
| Bone | 3 (5) |
| Adrenals | 2 (3) |

### Statistical methods

Using standard statistical methods, a two-stage design was employed in the protocol (Gehan, 1961). If no CR or PR were noted in the first 14 patients, a response rate of > 20% could be excluded with 95% confidence and accrual would stop. If at least one CR or PR was observed, > 30 patients were to be entered in the study to determine the response rate more accurately. For the response rates, 95% confidence intervals (CI) were calculated as previously described (Anderson et al, 1982). The distribution of TTP and time to death from the date of study entry were estimated using the Kaplan–Meier product-limit method (Kaplan and Meier, 1958).

### RESULTS

### Patient population

Between November 1995 and March 1997, a total of 70 patients were entered onto this trial from five different institutions. Only four patients were ineligible by study criteria. One had a history of cardiac impairment, two had inadequate baseline documentation of measurable disease, and one had acinar cell instead of adenocarcinoma histology. All other patients were considered evaluable for response and toxicity assessment. The demographic data, prior surgical procedures, histological grade and sites of metastatic tumour of the 66 eligible patients are listed in Table 1. There were 39 men and 27 women, with a median age of 62 years. Fourteen had undergone prior potential curative surgery with disease recurrence after a median of 10 months (range 3–76). Fifteen patients had palliative bypass surgery for biliary and/or gastric decompression, and eight patients had received endoscopic stents for relieving obstructive jaundice before study entry. The large majority of patients had multiple intra-abdominal sites of metastases, and all except six patients were suffering from disease-related symptoms: 51 of the 60 symptomatic patients (85%) had pain at study entry, 32 of whom (63%) had a baseline pain intensity score greater than 20 points, and 46 (90%) required more than 10 morphine-equivalent mg day$^{-1}$ for control of pain. Similarly, most patients had an impaired performance status at study entry (91%), and 48 (73%) had experienced weight loss, ranging from 5% to 37% of premorbid body weight.

### Treatment summary

A total of 271 cycles were administered to the 66 patients with a median of 4 cycles per patient (range 1–6). The median duration of treatment was 128 days, with a range of 28–168 days. Treatment was stopped because of toxic side-effects in only one patient, two warranted early discontinuation for other, personal reasons, and in all other patients therapy was stopped because of progression, including six patients with tumour complications while still receiving chemotherapy, who required palliative endoscopic or surgical intervention (four biliary and two intestinal obstructions). There were no major protocol violations.

### Objective response and survival

Response, time to progression and survival data are summarized in Table 2. The overall response rate was 21% for all 66 eligible patients (95% CI 12–33%), including one CR and 13 PR. The median time to response was 2.7 months (range 1.8–4), and the median duration of response was 7.5 months (range 3–22). An additional 27 patients (41%) showed stabilization of disease lasting for a median of 5.8 months (range 3–13.5) and in 25 patients (38%) tumour progression could not be abrogated by chemotherapy.

At the time of this analysis, all patients had experienced progressive disease. Fifty-seven patients (86.4%) have died, and

© 1999 Cancer Research Campaign 

**Table 2** Summary of treatment results (*n* = 66)

| | |
|---|---|
| Complete response | 1 (1.5%) |
| Partial response | 13 (20%) |
| Stable disease | 27 (41%) |
| Progression | 25 (38%) |
| Overall response rate | 14/66 (21%) |
| 95% confidence interval | 12%–33% |
| Time to progression (months) | |
| Median | 3.8 |
| Range | 1.5–23.0 |
| Overall survival (months) | |
| Median | 7.8 |
| Range | 1.5–28.0+ |
| 1-year survival rate | 21% |

the median follow-up duration of the nine patients still alive is 12 months. The median time to progression was 3.8 months (range 1.5–23). Median survival was 7.8 months (range 1.8–28+), and the probability of surviving beyond 12 months was 21.2%.

### Clinical benefit response

Sixty patients with tumour-related symptoms (pain and/or impaired performance status ± weight-loss) were considered evaluable for clinical benefit response. In 15/51 patients suffering from pain at study entry, pain intensity and/or analgesic use was reduced compared to baseline values, and 30 were classified as stable in this category (including 8/9 patients without pain at entry, but ≥ 1 other specific cancer-related symptom). Improvement in pain with no worsening of performance status occurred in nine patients, whereas both pain and performance status improved in six. An additional ten patients had an improvement in performance status while being rated stable in the pain category. Therefore a total of 25 patients were classified as clinical benefit responders by primary measures. With regard to weight gain, the secondary measure of clinical benefit, eight patients had a positive change (> 7% increase from baseline). Seven of these patients had already improved in one of the primary measures, and one was considered stable in pain and performance status. According to this case, the total number of primarily symptomatic patients experiencing a clinical benefit response with gemcitabine + epirubicin + G-CSF increased to 26 (43.3%). The median time to achieve a clinical benefit response was 7 weeks, and the median duration of clinical benefit was 22 weeks.

### Toxicity

All 66 patients, who received a total of 271 cycles of therapy (813 administrations of gemcitabine), were assessable for toxicity. Side-effects associated with treatment are listed in Table 3. The dose-limiting toxicity was myelosuppression. Leukopenia occurred in 57 patients (86%), and was grade 3 or 4 in 22 patients (33%). The median nadir WBC count was 3430 $\mu l^{-1}$ (range 500–25 900 $\mu l^{-1}$). The time to WBC count recovery to more than 3000 $\mu l^{-1}$ was short, i.e. 96% of episodes of leukopenia resolved within 7 days. The variations in granulocyte counts paralleled those of WBCs, and the median nadir count was 1759 $\mu l^{-1}$ (range 60–10 130 $\mu l^{-1}$). Thrombocytopenia was noted in a total of 35 patients (53%), and was grade 3 or 4 in seven and four patients respectively. There were no episodes of bleeding. The median nadir platelet count was 122 000 $\mu l^{-1}$ (range 9000–968 000 $\mu l^{-1}$) with no evidence of a cumulative nature of this side-effect. Only four patients (6%) developed grade 3/4 anaemia requiring RBC transfusion, whereas mild anaemia was recorded in 48 patients (73%). The median nadir of haemoglobin was 10.6 g $dl^{-1}$ (range 5.4–13.7 g $dl^{-1}$). Eleven patients developed documented infection, and two of them required hospitalization for granulocytopenic sepsis, both of whom were treated successfully.

Minor treatment-related elevations in liver functional parameters were noted in fewer than one-third of the patients, and did not result in any dose modifications or discontinuation from treatment.

Apart from hair loss in 79% (total alopecia 23%), gastro-intestinal toxicities were the most frequently encountered non-haematologic side-effects: nausea/vomiting occurred in 32%, though symptoms were generally mild, confined to the day of drug administration, and responsive to standard anti-emetic therapy.

**Table 3** Summary of maximum treatment-associated toxicities (*n* = 66)

| Toxicity | Number of patients/WHO toxicity grade (%) | | | |
|---|---|---|---|---|
| | 1 | 2 | 3 | 4 |
| Haematological and other laboratory-based toxicity | | | | |
| Leukopenia | 17 (26) | 18 (27) | 19 (29) | 3 (5) |
| Granulocytopenia | 13 (20) | 15 (23) | 19 (29) | 9 (14) |
| Thrombocytopenia | 15 (23) | 9 (14) | 7 (11) | 4 (6) |
| Anaemia | 22 (33) | 26 (39) | 3 (5) | 1 (2) |
| Bilirubin | 4 (6) | 2 (3) | – | – |
| Alkaline phosphatase | 15 (23) | 6 (9) | 1 (2) | – |
| Serum transaminases | 14 (21) | 8 (12) | 2 (3) | – |
| Symptomatic toxicity | | | | |
| Nausea/vomiting | 15 (23) | 4 (6) | – | |
| Stomatitis | 3 (5) | 5 (8) | – | – |
| Diarrhoea | 4 (6) | 2 (3) | – | – |
| Constipation | 3 (5) | 4 (6) | 2 (3) | – |
| Infection | 5 (8) | 4 (6) | 2 (3) | – |
| Fever | 5 (8) | 2 (3) | – | – |
| Alopecia | 14 (21) | 23 (35) | 15 (23) | – |
| Cutaneous | 7 (11) | 3 (5) | – | – |
| Phlebitis | 3 (5) | 2 (3) | – | – |

 *© 1999 Cancer Research Campaign*

Stomatitis was recorded in eight patients, and diarrhoea or constipation occurred in six and seven patients respectively. Uncommon non-myelosuppressive toxicities included minor (grade 1 or 2) skin rash (15%) that was treated symptomatically with topical corticosteroids and/or systemic antihistamines, fever in the absence of infection (11%), chemically-induced phlebitis (8%), peripheral neuropathy (3%) and G-CSF-related myalgias/arthralgias and/or fever in 3%.

Twenty patients (30%) had at least one treatment delay of 1 week at some time during therapy, and the total of delayed courses was 32 (12%). The reasons for delayed courses were haematologic in 16 and non-haematologic in five, including protracted stomatitis, intercurrent infection, port-a-cath-implantation, palliative surgery and personal reasons in one patient each.

Eighteen patients (27%) had a 25% dose reduction of cytotoxic drugs during treatment according to the study protocol, because of severe haematologic ($n$ = 13) or other systemic toxicities ($n$ = 2), or both ($n$ = 3). Only one patient discontinued therapy because of toxicity (protracted thrombocytopenia for > 2 weeks), and there were no toxic deaths. Overall, there was no evidence for cumulative toxicity, since both treatment delays and requirements for dose reductions were not more common during late cycles.

### DISCUSSION

Although in a randomized trial the novel cytidin analogue gemcitabine was shown to be more effective than 5-FU in advanced pancreatic cancer, the reported objective response rate was only 5.4%, there was only a modest survival advantage (5.65 vs 4.41 months), and only one out of four patients (23.8%) experienced clinical benefit (Burris et al, 1997). Further improvements are certainly warranted, and might be achieved by combining gemcitabine with other active cytotoxic drugs. Encouraging preliminary data in patients with this common malignancy have been reported very recently for its combination with cisplatin (Heinemann et al, 1997), as well as bolus (Cascinu et al, 1998) and continuous infusion 5-FU (Cortes-Funes et al, 1998). Epirubicin is another classical agent that has shown to be active for the treatment of advanced pancreatic cancer with a different mode of action and a toxicity profile that is distinct from that of gemcitabine (Wils et al, 1985; Kornek et al, 1995). A phase I/II combination study with this anthracycline using 1000 mg $m^{-2}$ of gemcitabine and 20 mg $m^{-2}$ of epirubicin on days 1, 8 and 15 of a 28-day cycle that has been performed in patients with advanced breast cancer, has demonstrated feasibility, potential synergistic activity and acceptable tolerance with neutropenia constituting the dose-limiting toxicity (Lueftner et al, 1996). The aim of the present study was to determine the anti-tumour activity of a comparable drug dose regimen in advanced pancreatic adenocarcinoma, though the entire dose of the anthracycline was given on day 1, followed by a short, i.e. a 5-day course of the haematopoetic growth factor G-CSF in order to counteract/minimize myelosuppression.

In this study we obtained a 21% overall remission rate (95% CI, 12–33%) in 66 evaluable patients and a median response duration of 7.5 months. With an additional 41% of patients experiencing stable disease (for a median duration of 5.8 months), chemotherapy with gemcitabine, epirubicin and G-CSF resulted in abrogation of progression of this aggressive tumour in almost two-thirds. These objective response data are even more interesting considering the fact that all of our patients had metastatic disease, as opposed to most other studies in pancreatic cancer that have also included patients with only advanced locoregional disease, who are known to have a much better prognosis (Warshaw and Fernandez-del Castillo, 1992; Andre et al, 1996). Keeping this in mind, the most striking results of our study are the median time of progression-free (3.8 months) and overall survival (7.8 months), as well as the frequent palliative effects obtained: clinically significant and sustained improvements in pain, analgesic consumption and/or Karnofsky performance score were observed in 43% of symptomatic patients, which in agreement with the objective results of treatment, is almost a doubling of the rate of clinical benefit responders reported for gemcitabine alone (using the same rigorous definitions). The onset of clinical benefit (7 weeks) was equally rapid as reported by Burris et al (1997) and its duration was 22 weeks. It seems noteworthy that the beneficial effects of gemcitabine + epirubicin + G-CSF were not negated by more frequent or severe clinically relevant treatment-related toxicities. Although grade 3 and 4 neutropenia was more commonly observed than in the gemcitabine trial with previously untreated patients (43% vs 23%), it was rarely associated with serious infections, a finding that is likely to be related to the prophylactic use of a haematopoetic growth factor. Thrombocytopenia was also more pronounced with the combination regimen, but again there were no (bleeding) complications and/or requirement for platelet substitution. As it concerns the frequency and degree of non-haematologic adverse reactions, except for alopecia the addition of epirubicin to gemcitabine did not seem to result in an increase when compared to historical data of gemcitabine monotherapy. The even lower rate of severe gastrointestinal toxicities (< 5% in the present trial) might be explained by routine concomitant administration of a serotonin antagonist with chemotherapy (plus additional corticosteroids on the day of epirubicin).

In conclusion, the combination of gemcitabine + epirubicin + G-CSF seems to be an effective palliative therapy for non-pretreated advanced pancreatic cancer accompanied by acceptable toxicity. Although objective and clinical benefit response as well as survival data suggest a possible advantage over gemcitabine monotherapy, results will have to be confirmed in a randomized trial.

### ACKNOWLEDGEMENTS

This study was supported in part by the Austrian Cancer Society/Section of Niederoesterreich and the ‘Gesellschaft zur Erforschung der Biologie und Behandlung von Tumorkrankheiten’.

### REFERENCES

Anderson JR, Bernstein L and Pike MC (1982) Approximate confidence intervals for probabilities of survival and quantiles in life-table analysis. *Biometrics* **38**: 407–416

Andre T, Lotz JP, Bouleuc C, Azzouzi K, Houry S, Hannoun L, See J, Esteso A, Avenin D and Izrael V (1996) Phase II trial of 5-fluorouracil, leucovorin and cisplatin for the treatment of advanced pancreatic adenocarcinoma. *Ann Oncol* **7**: 173–178

Burris III HA, Moore MJ, Andersen J, Green MR, Rothenberg ML, Modiano MR, Cripps MC, Portenoy RK, Storniolo AM, Tarassoff P, Nelson R, Dorr A, Stephens CD and Von Hoff DD (1997) Improvements in survival and clinical benefit with gemcitabine as first-line therapy for patients with advanced pancreas cancer: a randomised trial. *J Clin Oncol* **15**: 2403–2413

Cascinu S, Silva RR, Barni S, Labianca R, Frontini L, Pancera G, Zaniboni A, Ferretti B, Giordani P, Pessi MA, Fusco V, Luporini G, Catalano G and Cellerino R (1998) Gemcitabine (GEM) and 5-fluorouracil (5FU) in advanced

© 1999 Cancer Research Campaign pancreatic cancer: a GISCAD phase II study. *Proc Am Soc Clin Oncol* **17**: 264a (abstract)

Casper ES, Green MR, Kelsen DP, Heelan RT, Brown TD, Flombaum CD, Trochanowski B and Tarassoff PG (1994) Phase II trial of gemcitabine (2′2′-difluorodeoxy-cytidine) in patients with adenocarcinoma of the pancreas. *Invest New Drugs* **12**: 29–34

Cortes-Funes H, Castellano D, Paz-Ares L, Gravalos C, Alonso S, Hitt R and Hidalgo M (1998) A phase I–II study of gemcitabine (GEM) plus continuous infusion (CI) 5-fluorouracil (5-FU) in patients with advanced pancreatic cancer (APC). *Proc Am Soc Clin Oncol* **17**: 265a (abstract)

Garcia-Conde J, Lluch A, Perez-Manga G, Palomero I, Alba E, Rueda A, Moreno-Noguiera, Calvo E, Tarazona Y and Lopez-Martin E (1997) Gemcitabine + doxorubicin in advanced breast cancer: final results from an early phase II study. *Proc Am Soc Clin Oncol* **16**: 147 (abstract)

Gehan EA (1961) The determination of the number of patients required in a follow-up trial of a new chemotherapeutic agent. *J Chron Dis* **13**: 346–353

Heinemann V, Wilke H, Possinger K, Mergenthaler K, Clemens M, König HJ, Illiger HJ, Lackhoff A, Blatter J, Schallhorn A and Fink U (1997) Activity and tolerability of gemcitabine plus cisplatin in advanced metastatic pancreatic carcinoma. *Eur J Cancer* **33**: 274 (abstract)

Hertel LW, Boder GB, Kroin JS, Rinzel SM, Poore GA, Todd GC and Grindey GB (1990) Evaluation of the antitumor activity of gemcitabine (2′2′-difluoro-2′-deoxycytidine). *Cancer Res* **50**: 4417–4422

Kaplan EL and Meier P (1958) Non-parametric estimation from incomplete observations. *J Am Stat Assoc* **53**: 458–481

Kornek G, Raderer M, Schenk T, Pidlich J, Schulz F, Globits S, Tetzner C and Scheithauer W (1995) Phase I/II trial of dexverapamil, epirubicin, and granulocyte-macrophage-colony stimulating factor in patients with advanced pancreatic adenocarcinoma. *Cancer* **76**: 1356–1362

Lüftner D, Grunewald R, Flath B, Akrivakis C, Mergenthaler HG, Blatter J and Possinger K (1996) Gemcitabine with dose escalated epirubicin in advanced breast cancer: results of a phase I study. *Ann Oncol 7*: **17** (abstract)

Miller AB, Hoogstraten B and Staquet M (1981) Reporting results of cancer treatment. *Cancer* **147**: 207–214

Parker SI, Tong T and Bolden S (1996) Cancer statistics 1996. *CA Cancer Clin J* **65**: 5–27

Ribas A, Albanell J, Bellmunt J, Sole-Calvo LS, Bermejo B, Gallardo E, Vidal R, Vera R, Eres N, Carulla J and Baselga J (1996) Five-day course of granulocyte colony-stimulating factor in patients with prolonged neutropenia after adjuvant chemotherapy for breast cancer is a safe and cost-effective schedule to maintain dose-intensity. *J Clin Oncol* **14**: 1573–1580

Rothenberg ML, Moore MJ, Cripps MC, Andersen JS, Portenoy RK, Burris HA III, Green MR, Tarassoff PG, Brown TD, Casper JS, Storniolo AM and Von Hoff DD (1996) A phase II trial of gemcitabine in patients with 5-FU refractory pancreas cancer. *Ann Oncol* **7**: 347–353

Schnall SF and Macdonald JS (1996) Chemotherapy of adenocarcinoma of the pancreas. *Semin Oncol* **23**: 220–228

Warshaw AL and Fernandez-del Castillo C (1992) Pancreatic carcinoma. *N Engl J Med* **326**: 455–465

Wils J, Bleiberg J, Blijham G, Dalesio O, Duez N, Lacave A and Splinter T (1985) Phase II study of epirubicin in advanced adenocarcinoma of the pancreas. *Eur J Cancer Clin Oncol* **21**: 191–194

*© 1999 Cancer Research Campaign*
